# Exploring community perceptions in preparation for a randomised controlled trial of biofortified flour in Pakistan

**DOI:** 10.1186/s40814-020-00664-4

**Published:** 2020-08-18

**Authors:** Usman Mahboob, Heather Ohly, Edward J. M. Joy, Victoria Moran, Mukhtiar Zaman, Nicola M. Lowe

**Affiliations:** 1 Khyber Medical University, Phase V, Hayatabad, Peshawar, Khyber Pakhtunkhwa Pakistan; 2 University of Central Lancashire, Preston, Lancashire PR1 2HE UK; 3 London School of Hygiene & Tropical Medicine, Keppel Street, London, WC1E 7HT UK; 4 University of Central Lancashire, Preston, Lancashire PR1 2HE UK; 5 Rehman Medical College, 5-B/2, Phase-V, Hayatabad, Peshawar, Khyber Pakhtunkhwa Pakistan

**Keywords:** Biofortification, Zinc, Iron, Deficiency, Community, Pakistan, Formative, Qualitative, Adolescent girls

## Abstract

**Background:**

Biofortification of staple food crops may be a cost-effective and sustainable approach to reducing micronutrient deficiencies in resource-poor settings with low dietary diversity. However, its success depends on uptake by the local population. This paper presents formative research conducted in a remote, rural community in North West Pakistan, prior to commencing a randomised controlled trial to test the effectiveness of consuming zinc-biofortified wheat flour for alleviating zinc deficiency. It explored local community members’ knowledge, understanding and attitudes towards biofortification and views on members of their community taking part in the trial.

**Methods:**

Four focus group discussions were conducted with male and female community members (separately for cultural reasons) and four in-depth interviews were conducted with Jirga members—respected male elders. Participation was limited to households that were ineligible for the trial so that we could explore the perspectives of community members who were not influenced by the incentives of the trial. Focus group participants were selected at community events for transparency. Data collection took place at the local school and homes of Jirga members. Thematic analysis was undertaken, using a combination of deductive and inductive approaches to identify key themes.

**Results:**

A total of 47 men and women participated in this study. Participants reported clear motivation to access and consume more nutritious flour, believing this would bring health benefits, particularly to women and children. Trusted members of the local community, including Jirga members and female health workers, should be involved in providing information on biofortified flour (and the trial) to increase levels of awareness and acceptance. Without their involvement, there is a risk that biofortified flour would be mistrusted. The cost of flour is the main factor affecting purchasing decisions, and biofortified flour will need to be cost-competitive to achieve widespread uptake in marginalised, rural communities.

**Conclusion:**

This formative study generated rich, qualitative data from a range of community stakeholders to improve the understanding of important barriers and facilitators to the widespread acceptability and adoption of biofortified wheat. Implementation research such as this will inform future decision-making in relation to scaling up biofortified wheat in Pakistan.

## Background

Zinc and iron deficiencies are a global public health problem, with the greatest burden occurring in low and middle-income countries. In Pakistan, the National Nutrition Survey in 2018 indicated that 22.1% of women of reproductive age (WRA) and 18.6% of children under 5 were zinc deficient; likewise, 18.2% of WRA and 28.6% of children under 5 had iron deficiency anaemia [1]. The prevalence of zinc and iron deficiencies is likely to be even higher in low-resource, rural communities where diets are dominated by plant source foods (with lower concentrations and bioavailability of these micronutrients). Zinc and iron deficiencies have negative long-term consequences for health, particularly amongst women of childbearing age, adolescents and children, and it is imperative that sustainable and cost-effective solutions are found.

An increasing body of evidence suggests that biofortification, a process by which the nutritional quality of food crops is improved through plant breeding and agronomic practices, may be a cost-effective and sustainable approach to reducing micronutrient deficiencies [2]. The HarvestPlus programme in collaboration with the National Agricultural Research Centre, Pakistan, has developed a variety of wheat (Zincol-2016) with significantly greater zinc and iron concentrations compared to standard varieties. Wheat is the staple crop in Pakistan and most families consume chapatis (made from wheat flour) with every meal. Therefore, biofortified flour may benefit communities who cannot afford to consume a diverse range of foods.

BIZIFED2 (Biofortification with zinc and iron for eliminating deficiency in Pakistan) is the first large-scale investigation into the potential of biofortified wheat to reduce zinc and iron deficiencies amongst adolescent girls and children in Pakistan. We are conducting a double-blind, cluster randomised controlled trial (RCT) to test the effectiveness of consuming biofortified flour for alleviating zinc and iron deficiencies (registration number: ISRCTN17107812). Participating households include an unmarried adolescent girl (aged 10-16 years) and a child (aged 1-5 years). The RCT involves two phases of 6 months duration (12 months in total). In phase 1, households will be provided with locally purchased non-biofortified flour. In phase 2, households will be paired and randomised to either the control or intervention arm of the study. The control arm will continue to be provided with locally purchased non-biofortified flour, whilst the intervention arm will be provided with flour milled from locally grown biofortified wheat (Zincol-2016).

In addition to the efficacy of alleviating zinc and iron deficiencies through consumption of biofortified flour, the success of biofortification ultimately depends on uptake by the local population. Therefore, one of the aims of BIZIFED2 is to explore the socio-cultural factors and market systems that affect the sustainable uptake of biofortified wheat in Pakistan. This implementation component of our research will be conducted in partnership with local stakeholders (including community members, wheat farmers and flour millers) and will inform future decision-making in relation to scaling up biofortified wheat in Pakistan.

This paper presents a formative research study conducted prior to commencing the BIZIFED2 RCT. The study objectives were to explore local community members’ knowledge, understanding, and attitudes towards biofortification and views on members of their community taking part in a research study.

## Methods

### Study setting

A remote, rural community situated 25 km southeast of the city of Peshawar, Khyber Pakhtunkhwa (KP) Province, North West Pakistan. This area is the former Frontier Region Peshawar (FR Peshawar) federally administered tribal area (FATA), which merged with KP in 2018. Most households earn less than USD 10 per day from small-scale agriculture, government and semi-government employments, and manual labour in the nearby brick kilns. The dominant religion is Islam and the dominant ethnic group is Pashtun. The government of Pakistan established a refugee camp in this area for Afghan refugees during the Soviet-Afghan War in the 1980s. This was our first study in this community, but we previously conducted research in a neighbouring community as part of our foundation study on biofortification [3, 4].

### Study design

Formative research was conducted in August and September 2019, prior to the start of the RCT in November 2019. Qualitative methods were co-developed with local researchers and the community to ensure cultural appropriateness. Their insights into the local culture were invaluable to develop socially acceptable methods for recruitment, informed consent, and data collection (including careful refinement and translation of questions and probes). Four focus group discussions (FGDs) were conducted with male and female community members separately (two with men; two with women). Four in-depth interviews (IDIs) were conducted with Jirga members—respected male village elders. Participation was limited to households that were ineligible for the RCT so that we could explore the perspectives of community members who were not influenced by the possibility of receiving free flour for 12 months.

### Participants

#### Recruitment of male and female community members

The study area was divided into 10 clusters of households and five of those clusters were randomly selected (using the Open EPI software). A cluster mapping exercise found that 162 households in the five clusters were ineligible for the RCT (out of 231 households in total). From these 162 households, 60 households were randomly selected (using the Open EPI software) and the male heads of households were invited to attend a community event at the local school if they wished to participate in FGDs. At this event, 26 names were drawn from a bowl in a transparent selection process (aiming for up to 10 in each FGD and allowing for some dropouts). Eligible participants were aged over 16, residents in the community for at least year, willing and able to give informed consent and from households that were ineligible for the RCT. The same selection process was repeated for women, with another 60 out of 162 households randomly selected and a similar community event organised. It was culturally appropriate to conduct separate selection events for men and women.

#### Recruitment of Jirga members

Community mobilisers employed to support this project were asked to identify male elders who regularly attended Jirga meetings. A list was compiled of the most commonly mentioned names. Four Jirga members were randomly selected from this list (using the Open EPI software) and invited to participate in IDIs.

### Consent

Participants were provided with a study information sheet in the local language (Urdu) and this was read aloud to illiterate participants. Participants were asked to provide consent by marking initials or an X onto the consent form. The school principal acted as an independent witness to the consent process. Each participant was allocated a unique ID at recruitment, which was subsequently used on consent forms and all other documentation to preserve anonymity. At the start of each FGD or IDI, the researcher reminded all participants of the purpose of the study and their right to withdraw at any time. Study participants were not offered incentives or compensation because the research team agreed with the Jirga that financial support would be received at the community level during BIZIFED2 (such as enhanced health centre capacity and training of community health workers).

### Data collection

Topic guides were developed collaboratively by the research team and refined after feedback from the Jirga (Appendix). Questions explored participants’ awareness of micronutrient deficiencies and biofortification, usual flour purchasing patterns, considerations around buying biofortified flour, trusted sources of information in the community, views on the participation of adolescent girls in the trial and culturally appropriate ways to communicate with study participants and the wider community. Fieldwork was conducted by two local researchers (one male; one female) employed by the Abaseen Foundation—a charity that has been working in KP for over 20 years to deliver health, education and research projects. Both researchers had previous experience of conducting qualitative research and received specific training for this project in July 2019. FGDs with men and women were held on separate days at the local school. The FGDs were facilitated by one researcher (the same gender as the participants) and observed by the other researcher. The IDIs with Jirga members were conducted by the male researcher in the participant’s home or at the local school. All FGDs and IDIs were audio-recorded using a password-protected device.

### Data analysis

Audio recordings were transcribed and translated into English by a professional agency in the UK. Participants’ names were removed from the transcripts to maintain confidentiality. The English transcripts were checked for accuracy by one of the research team (UM). Thematic analysis was undertaken after a period of familiarisation and immersion with the data. Coding was completed manually by two members of the research team (UM/HO) and checked by two other members (EJ/VM). A combination of deductive and inductive approaches was used [5, 6]*.* For the deductive coding, a template (or codebook) was developed based on the study objectives and preliminary scanning of the text before commencing in-depth analysis of the data [7]. Four broad code categories formed the codebook (encompassing community members’ knowledge, understanding and attitudes towards biofortification and views on members of their community taking part in a research study) against which initial deductive coding was conducted. Additional codes were created and refined as the analysis progressed and these were data-driven (inductive approach). The codes from FGDs and IDIs were triangulated and key themes were identified. All authors were involved in reviewing and interpreting key themes during regular team meetings (online and face-to-face).

## Results

### Participant characteristics

A total of 47 men and women participated in this study during August and September 2019: 43 community members (17 men and 26 women) took part in four FDGs and four male Jirga members participated in IDIs. FGDs consisted of 7-13 participants and lasted between 41 and 59 min, whereas IDIs lasted between 18 and 28 min.

### Study findings

Six key themes were identified: (i) awareness of nutritional deficiency in the community; (ii) affordability and purchasing patterns; (iii) perceived benefits of the study; (iv) trust relations in the community; (v) suspicions and rumours; (vi) cultural perspectives on study methods. These themes are presented below and supported by illustrative quotes.

### Awareness of nutritional deficiency in the community

Jirga members were concerned about the health of their community and they linked this to poor diet and nutritional deficiency.

there are many of our children who are weak in terms of food and due to that the state of our children is very bad. (IDI Jirga 1)

Our area is poor; therefore, we do experience these deficiencies. Every sort of deficiency, there are sick people, it’s a poor area. Such food is not available nor do the people have businesses to be able to afford to eat fruit and other things and that’s why there are serious deficiencies. (IDI Jirga 4)

Health problems are compounded by poverty, where visits to doctors and resulting treatments have a significant impact on already restricted finances.

There are many children who are weak, there are females who have illness that we don’t have any idea of until it deteriorates and then we take them to doctor. At that point sometimes it is treated and sometimes not. But even if it cures, it entails a lot of expenses. (IDI Jirga 1)

Community members had some awareness of nutritional challenges, but some were unsure of the specific deficiencies affecting their community.

We don’t know it at all, we don’t know what nutrition deficiency is, what we know is that there is a deficiency. (FGD Women 1)

… but the uneducated people, they say that it will affect your mind, if there is a deficiency your bones will ache; you don’t have this vitamin or this thing otherwise, I don’t know either (FGD Men 2)

Others described specific deficiencies that were affecting their families, such as calcium deficiency and anaemia, and their concerns about the resulting impact on health.

In our area, people like wheat but there is a lot of calcium weakness in our children, all our women are weak and ill, this is a very poor area some have anaemia … We take tablets and medication. (FGD Women 1)

I have three daughters. One of my daughters is well but two of them have calcium deficiency. This deficiency makes me upset. There is a lot of deficiency in our children, very much. (FGD Women 1)

### Affordability and purchasing patterns

Ground flour tends to be more expensive than wheat, therefore some people prefer to stock up on wheat and take it to be ground at the local mill.

people are poor around here, when they have a little money, some are borrowing money, so we buy this because sometimes the bags of flour are too expensive. So, for that reason, we would have already stocked wheat and then we take it to be turned into flour, and we eat it, just for this reason. The bags [of flour] are not cost-efficient for us. (IDI Jirga 1)

There is often limited choice of flour in the marketplace, and it may not be possible to determine the quality of the flour.

what does it have to do with quality? It is just a bag and we don’t know what type of flour it contains; we just tell them to give us a bag of good flour. He then calculates it and we buy however many bags we need. (IDI Jirga 2)

White flour and expensive flour were assumed to be of higher quality. However, this Jirga member felt that price would be the most important factor for most people.

People buy the cheaper one too, but they look for good flour; flour that is white and also expensive, so people pay more attention to good flour. (IDI Jirga 2)

People negotiate individual payment arrangements with local shopkeepers. Those with regular jobs might prefer to pay monthly, whereas others would need to be more flexible.

What most people do is that some are teachers and so on, and they go monthly, and they have dealings with people. There are many shops, there are local shops and also refugees’ shops, they mostly buy it from their own people on a monthly basis and when they receive their salaries, they make payments. (IDI Jirga 4)

We have a deal with a particular shopkeeper, so in case there is no money then we can buy it on loan or when we have cash, we pay in cash; we have deals with shopkeepers. (IDI Jirga 2)

### Perceived benefits of the study

All participants had been made aware of the forthcoming BIZIFED2 RCT prior to the FDGs and IDIs through community engagement activities. Although they knew that their households would not be eligible to participate in the RCT, they were hopeful that the whole community would benefit from consuming biofortified wheat in the future.

All our women, our men, our seniors, all hope to receive wheat which is good for our health and if we receive wheat that completes the shortage in our children, we will be very happy about that. It is very beneficial for us and we wish that we receive such wheat which can reduce the illnesses of us poor people. (FGD Women 1)

They anticipated health benefits for future generations.

We feel that a young, unmarried girl would become healthy and fit, children will improve, and they will all pay their attention to education and they will be able to focus on education, the weakness they have got will be sorted out. Their weakness if any would be resolved. Children’s mental ability and understanding and learning ability will improve. When they get married and when they have children, it will be smooth and the children will be fit, their weakness will be gone. (FGD Women 1)

In addition to any perceived health benefits, Jirga members highlighted the financial advantage for households participating in the RCT, as they would receive free flour for 12 months.

Nobody should do anything without seeing any benefit in it. If there are no other advantages, at least they will get the flour. (IDI Jirga 3)

Because illness will be reduced, additionally they are poor people, if they receive flour it is a benefit for them. So, there is a double benefit, one is filling their stomachs and the other is dealing with illness. (IDI Jirga 4)

### Trust relations in the community

Jirga members identified themselves as trusted leaders and role models in the community.

When there is an issue then I gather them, then I tell them this issue is like this then they do not say no I’m not accepting this, then whatever I do, my whole village do it. When I do this thing, then my village will definitely do it. (ID Jirga 1)

Whatever the elders or the local Jirga people say, that’s what people listen to. (IDI Jirga 2)

They felt it was part of their role to raise awareness about the study and provide reassurance to the community when needed.

When I am informed about this project then I will convey it to all the villagers, women and children, telling them there is no harm in this project; it is for our benefit … The village elders will convey this message. (IDI Jirga 2)

Teachers are also seen as influential and trusted members of the community. School teachers have a broad reach because children pass on information to their families.

Sir, as far as I think, they will mostly understand the Masters group the most, and those people can convey it in a very positive way, they can make people understand … because everybody’s children go to them, so they can influence the children’s minds and those children can then take the message back to their families, so it can easily create a positive mindset. (IDI Jirga 3)

Other respected members of the community include medical doctors and religious leaders; people tend to follow their advice and guidance.

Here, everything is about seniors, secondly are the doctors’ opinion and when the doctor tells people that this thing is fine, and the doctor is also local, then people follow it properly. (IDI Jirga 1)

Obviously, there is an Imam of this area. He was there and he raised so it meant that everyone endorsed this issue as fine. Insha’Allah, such people can make them understand and they will. (IDI Jirga 3)

What you are saying has less impact on them; when [name of Islamic scholar] explains it, it has more impact. Most people will accept it from him. That is why they said to you that this should be done soon, and people should be reassured soon. If me and you were to reassure them, it will take time. If [name of Islamic scholar] was to reassure them, he can do it in one or two speeches. (FGD Men 1)

Overall, trust was placed in people who were perceived as local.

They are local people and they are after our interest, Insha’Allah. (IDI Jirga 1)

Furthermore, female community members said they trusted the female community health workers employed by the research project, who are themselves members of the local community.

The women who are visiting homes for this project, when they convey this message many people accepted it. When they came to our homes and they said it, we all believed in it and accepted it. What these women said, it has been accepted, people have accepted it. (FGD Women 1)

In contrast, participants felt that ‘outsiders’ would not understand the issues affecting their community and would not be understood by the local people.

Someone coming from outside will not be able to make them understand. (IDI Jirga 2)

### Suspicions and rumours

Participants explained that local people are often wary of foreign aid and NGOs. They warned that some people might spread rumours to dissuade people from participating in the BIZIFED2 study.

Those who do not take part will say these things; they will say that this is an NGO and so on and so forth; God knows what they have used in it; that they have done this to make sure people don’t have children you see. (FGD Men 2)

It is about perception and mentality; our people are very much uneducated. Before your women [research assistants employed on the study] had come and they spoke with our women and our females said that they were coming from America and said that if any problem comes from this, they are not responsible for this. So, in this way propaganda emerged. (IDI Jirga 2)

Concerns were raised about the possibility of harmful additives being added to the flour.

People would say that such and such things are mixed with it and they would spread rumours (IDI Jirga 4)

However, this would be less problematic if they knew the flour had been produced in Pakistan.

It should reach the public that this is a Pakistani product. (FGD Men 2)

This apparent mistrust of external organisations appears to have stemmed from community members’ experiences of previous interventions, such as the national polio vaccination campaign, which led to concerns about contamination and conspiracy.

It would be the same as the concerns they had about Polio, if anyone has such doubts, they might say that the method has been altered, it is very possible that someone would question it, absolutely. (IDI Jirga 3)

Other Jirga members, however, believed that the local people were less suspicious than in previous times and they are more likely to consider the potential benefits of participating in the study.

People do not have that negative mentality; people have improved now in comparison to the past. Now people do not have those wrong perceptions and thoughts. Nowadays everybody looks to question whether this thing is beneficial for me or not. So, whatever they find beneficial for them, they will go for it. (IDI Jirga 3)

### Cultural perspectives on study methods

Jirga members understood that blood sampling would be necessary in the study and would not harm the study participants (adolescent girls). However, they cautioned that some community members may not understand why this is necessary.

If I am going to be tested and my blood is taken for that, then taking blood will not make me ill or harm me. It is beneficial for me to find out whether this thing was useful for me or not. So, there is nothing wrong with taking blood at all. Testing blood is necessary whenever it is required. (IDI Jirga 1)

Then there will be doubt and suspicion that they gave us wheat, why are they taking our blood? Our people are very simple. You are taking it for test in order to improve our health, but they are simple people and they don’t understand. (IDI Jirga 2)

This elder compared blood sampling to injections, which have previously caused concerns amongst some community members—as mentioned above in relation to polio vaccinations.

Maybe some people question giving injections to children. They wonder what is in them, that maybe it can make people infertile [laughter]. Some people do raise such doubts and suspicions, so it’s possible that there would be some people who would say the same thing about this program as well. (IDI Jirga 3)

However, community members who participated in the FGDs did understand the reasons for blood sampling and felt that most parents would allow it once they understood.

I would say if they take the blood, we would test it. They will see it and check how it is; they say we will take blood and check it whether the zinc is enough or not or is it working with this wheat or not. (FGD Men 2)

The other thing is understanding and knowledge … When the people understand it; once the public understands it, who would want their daughter or son to have zinc deficiency? (FGD Men 2)

One male participant asked about the quantity of blood to be taken and suggested that some of the women in the community may be concerned about this.

How much blood will be taken? Because it has been taken and many women have become scared, like they take a lot of blood … They have become very scared you know; many women according to what I have heard in our village. (FGD 1 Men)

However, the female participants did not express any such concerns and were supportive of blood sampling (even when asked how they would feel if it was from their children).

If blood is taken from our children, it will become clear … If this test is done on our children, we don’t have any objection about it. We want this test to be done so that it is found out that [inaudible]. We don’t have any objection about it at all, blood can be taken from our children. We will bring them here to you, you take their blood. It is not an issue at all, it is good to find out what is the shortage [inaudible]. We will bring them to you, and you can take blood from them. (FGD Women 1)

Overall, both men and women were positive about the study and were not put off by the demands that would be placed on the community.

In my view, it is a very good thing that this research is done upon them, because there is weakness in our children’s bodies our aim is for our children’s health to improve, because my own children have the deficiency. I will become aware; there will be no harm so we have full confidence in you the whole Pakistan says that they should get this food; it is unclear whether it will resolve the deficiency or not, because my own suffers deficiency. (FGD Women 1)

## Discussion

### Implications of study findings

This formative research study explored the perceptions of a low-resource community in Pakistan, in relation to biofortified wheat and the feasibility of conducting a trial to test its effectiveness for alleviating zinc and iron deficiencies. This section provides an overview of the main study findings and how they informed the design and implementation of the RCT. It also highlights the relevance and transferability of our findings for future biofortification research studies and programmes to scale-up biofortified wheat in Pakistan and similar cultural contexts.

Study participants perceived that nutritional deficiencies are prevalent amongst women and children in the community, with calcium deficiency and anaemia cited as examples. These deficiencies were reported in the National Nutrition Survey in 2018, with higher rates in rural areas [1]. However, there may be less awareness of other micronutrient deficiencies such as zinc, iron, vitamin A and iodine, which are also common in rural areas of Pakistan.

Participants believed that availability of biofortified wheat would lead to health benefits for future generations of the community. There was clear motivation to make micronutrient-enriched flour available to families, which could translate into support of future studies or programmes that bring biofortified flour to the community. In terms of RCT implementation, we realised that clear communication would be important so that families did not assume there would be health benefits from consuming the biofortified wheat. A leaflet was produced to help the field team to explain the purpose of the study, that is, to establish whether there are health benefits associated with consuming biofortified wheat.

We found that the whiteness of the flour was perceived by participants to be an important indicator of flour quality. The micronutrient content of refined white flour may be the most important ‘target’ for biofortification programmes if they are to successfully increase dietary micronutrient intakes. Agronomic field studies suggest that zinc concentration can be increased in the starchy endosperm of wheat following application of foliar zinc fertilisers, which should translate into greater zinc concentrations in refined white flour [8].

Trusted figures in the community should be engaged to provide information on the purposes of the research study and ensure the community is informed about the study objectives. The early engagement with community leaders helped us to prepare for the RCT, with the aim of achieving high rates of participation and adherence to treatment (household consumption of the flour provided). Trusted people in this community include Jirga members, teachers, medical doctors, religious leaders and female community health workers. Importantly, information should be provided by locally known figures if the community is going to trust the messages. These findings highlight the importance of understanding the dynamics of social structures within a community before undertaking any research study.

Developing trust in biofortified wheat flour is critical to avoid suspicions and rumours. Previous studies in marginalised communities in Pakistan have evaluated attitudes towards biofortified wheat [4] and other health interventions such as iodised salt [9] and polio immunizations [10]. Notably, these studies also found that acceptance was contingent on addressing community fears and rumours. There is a tendency to view ‘foreign’ products with suspicion and respected, local stakeholders need to be engaged to allay these suspicions. In particular, the Jirga elders play a vital role in influencing the acceptability of new products to community members [11]. Similar engagement processes may also be relevant for biofortification programmes aiming to achieve widespread uptake of new crop varieties or flour products.

Affordability was perceived to be the most important factor influencing flour and wheat purchasing decisions. Some community members purchase wheat grain rather than flour, since this is a cheaper option. Another strategy is purchasing flour on credit from certain shopkeepers, whilst the refugee community sometimes purchase flour from others within their community. These findings are not surprising since previous research has shown that affordability was the greatest barrier to achieving a nutritious diet in Pakistan, with 67% of households unable to afford a nutritious diet [12]. Cost and purchasing patterns are likely to be important considerations for future biofortification programmes in Pakistan.

In relation to conducting scientific research with adolescent girls, participants commented that blood sampling may cause concerns in the community. Therefore, the need and the process for taking blood (and other tissue samples) should be clearly explained to study participants to avoid any issues. In this community, we undertook community engagement activities before and during the RCT, in which the actual volume of a blood sample was demonstrated to reassure participants and their families that we were not going to collect large volumes.

### Strengths and limitations

For any new biofortified crop, it is essential to understand the context into which the crop may be introduced. This includes local attitudes, awareness, acceptability and affordability of the crop and knowledge of local market systems. In combination with evidence of effectiveness for alleviating micronutrient deficiencies, this kind of contextual research may be used to support scaling up activities and innovations to promote sustainable uptake of the new crop. In the biofortification literature, stakeholder perceptions, consumer acceptance and willingness to pay have been assessed using a range of interdisciplinary methods, such as SWOT (strengths, weaknesses, opportunities and threats) analysis, sensory evaluation, experimental auctions and revealed choice experiments [13–15]. However, very few studies have conducted in-depth qualitative studies with local community stakeholders to identify relevant contextual factors and potential challenges prior to implementation.

A major strength of this study is the mixed-method approach, generating rich, qualitative data, including men, women and a range of stakeholders in the community.

A limitation of this study is the potential respondent bias. Some of the female study participants were unhappy because they knew that they would not be eligible for the RCT, which led to disinterest in focus group participation due to lack of incentive as they would not receive any flour. In future qualitative studies in this community, we will show our appreciation to participants with small and culturally appropriate thank you gifts (such as kitchen utensils or food items) to avoid feelings of dissatisfaction or resentment. Another limitation was the lack of suitable communal space to conduct the FGDs. The local school was deemed to be the most appropriate location, but this was not convenient for study members who lived further away. Hot weather made it more difficult for participants to attend the FGDs, which may have influenced their perceptions and responses. Audio data were recorded, transcribed and translated, but we did not supplement this with observational notes to capture body language and other non-verbal insights.

## Conclusions

Biofortification has the potential to alleviate certain micronutrient deficiencies amongst hard-to-reach, rural communities in Pakistan. Yet, there is little knowledge about the acceptability of biofortified wheat in these communities. We conducted formative research in advance of a trial that involves distribution and consumption of biofortified flour, to inform the implementation of the trial and to improve the understanding of important barriers and facilitators to the widespread acceptability and adoption of biofortified wheat. The community in the current study reported clear motivation to access and consume more nutritious flour, believing this would bring health benefits, particularly to women and children. However, the formative research also highlighted the need to engage with trusted members of the community, including Jirga members and female health workers, to provide the community with information about the planned trial objectives and activities and ensure acceptance by the community. Without this level of community involvement, there is a risk that biofortified flour would be mistrusted. Future research studies are likely to require similar approaches, as are programmes that promote biofortified crops or products, in order to address potential cultural barriers. The cost of flour is the main factor affecting purchasing decisions, and biofortified flour will need to be cost-competitive to achieve widespread uptake in marginalised, rural communities.

## Data Availability

The datasets used and analysed during the current study are available from the corresponding author on reasonable request.

## Appendix

FGD topic guide for formative study

Adult men/adult women/Jirga (interviews)

A focus group discussion (FGD) is a conversation between a homogenous or heterogeneous group facilitated by a moderator. The conversation gives different opinions on the topic under discussion. An FGD is used in situations where the topic of discussion is of common interest to the participants and does not have the issue of disclosure of sensitive information. Further, it is done when getting participants around one table is not difficult, or if the topic needs to be investigated in-depth through discussion to further explore it. The sample required for FGD is small due to the qualitative nature of the data. Usually, 4-10 people would participate in one FGD. The strength of an FGD is the discussion that explores the research topic in-depth. The limitations of an FGD are as follows: when there is any sensitive information that some participants may not like to share, or if one of the group members is dominating or if some of the group members are quiet and not expressing themselves. However, an experienced facilitator or moderator can overcome these issues.

The researcher can take help of another person for logistic support or s/he can assign different roles to the FGD participants. The roles in FGD are as follows: a ‘scribe’ who would take the notes, a facilitator or moderator who would steer the discussion, a time keeper who would keep the group to complete tasks within time, and participants who would fully participate in the discussion. Use of white board, flip charts and showing videos in some situations may also be part of an FGD. Moreover, an FGD guide is also required that can be sent to the study participants beforehand or just before starting the FGD. The guide includes introduction to the topic, why the participant has been selected, the total time that would be required, any incentives, and the guiding questions with prompts where relevant. Moreover, the moderator of FGD may have some additional details in her/his guide, on how to facilitate the session.

Reference: Mahboob U. (2018) Data Collection tools in qualitative and quantitative research. Ch. 9 In Book: Scientific writing: a guide to the art of medical writing and scientific publishing. Professional Medical Publication.

Questions relating to biofortification

1. Which type of flour does your household consume?
  *Probe why they select this type of flour, what influences their decisions?*
2. What do you understand by the term micronutrient deficiencies?
  *Probe general awareness (define micronutrients as vitamins and minerals) and explain that Pakistan has high levels of zinc and iron deficiency. No need to discuss causes and consequences in detail—say that micronutrients are important for childhood growth and development and long-term health outcomes.*
3. What do you understand by the term biofortification?
  *Probe awareness of how biofortified crops are developed, higher micronutrient content, pros/cons in comparison to GM crops etc.*
4. Would you consider buying biofortified flour if it was available locally?
  *Probe what they would want to know before they would buy it and any concerns. Note: community previously raised concerns about biofortified flour being a family planning intervention—do not mention unless they do.*
5. Who in your household buys the flour and where from?
  *Probe purchasing patterns, e.g. how often, what kind of shop?*
6. If these two types of flour [show pictures of sacks: one biofortified, same price] were available in your local market, which would you choose and why?
  *Probe reasoning—but do not suggest benefits or concerns yourself.*
7. Who would you seek advice from if you were unsure about biofortified flour?
  *Probe who they trust to provide sound advice in the community.*

Questions relating to research study

We will soon be recruiting participants from your community for our trial of biofortified wheat. Households with an adolescent girl (10-16 years) and a child (1-5 years) will be selected. Adolescence is a critical time for physical development, and an opportunity to improve girls’ health before they become mothers.

Selected households will receive free flour for 12 months during the trial. They will not know if the flour is biofortified or not—this will be randomised. Adolescent girls will be required to provide blood samples at various stages of the trial so that we can assess their zinc and iron status. They will also be interviewed about their daily food intake. Mothers of younger children will be interviewed about the child’s daily food intake and asked to report any diarrhoea and respiratory infections.

8. Have you ever participated in a research study before today?
  *Probe for details if they say yes—but we suspect unlikely.*
9. What are your feelings about adolescent girls as research participants?
  *Probe anyone who has an adolescent daughter how they would feel.*
10. Do you have any questions about the blood sampling?
  *Anticipate concerns about the amount of blood and how it will be used. They might mention previous health programmes etc. Reassure that blood samples will only be used to assess nutrient levels and data will be treated confidentially.*
11. Do you think households in this community will be willing to participate in the trial?
  *Probe about motivations and concerns—but do not suggest yourself.*
12. What would be the best way to communicate with members of the community about the purpose of this study?
  *Probe about the role of Jirga, health centre staff and female community health workers.*
13. How would you feel if your neighbor was selected and your household was not?
  *Opportunity to explain about the random selection process and community benefits.*
14. Do you have any other concerns or questions about the trial?
  *Thank you for participating in this focus group today.*

## Abbreviations

BIZIFED2: Biofortification with zinc and iron for eliminating deficiency in Pakistan
FATA: Federally administered tribal areas
FGD: Focus group discussion
IDI: In-depth interview
KP: Khyber Pakhtunkhwa
RCT: Randomised controlled trial
UK: United Kingdom
USD: United States dollars
WRA: Women of reproductive age

## References

[1] Government of Pakistan and UNICEF. National nutrition survey 2018: key findings report. https://www.unicef.org/pakistan/reports/national-nutrition-survey-2018-key-findings-report. Accessed 24 July 2019.

[2] Lockyer S, White A, Buttriss JL (2018). Biofortified crops for tackling micronutrient deficiencies – what impact are these having in developing countries and could they be of relevance within Europe?. Nutrition Bulletin..

[3] Lowe NM, Khan MJ, Broadley MR, Zia MH, McArdle HJ, Joy EJM (2018). Examining the effectiveness of consuming flour made from agronomically biofortified wheat (Zincol-2016/NR-421) for improving Zn status in women in a low-resource setting in Pakistan: study protocol for a randomised, double-blind, controlled cross-over trial (BiZiFED). BMJ Open..

[4] Ohly H, Joy EJM, Broadley MR, Khan MJ, McArdle HJ, Medhi R (2019). Exploring socio-cultural aspects of the food environment: study perspectives from Pakistan. UNSCN Nutrition..

[5] Braun V, Clarke V (2006). *Using thematic analysis in psychology*. Qualitative Research in Psychology..

[6] Fereday J, Muir-Cochrane E (2006). Demonstrating rigor using thematic analysis: a hybrid approach of inductive and deductive coding and theme development. International Journal of Qualitative Methods..

[7] Crabtree B, Miller W. A template approach to text analysis: developing and using codebooks. In: Crabtree & W. Miller (Eds.) Doing qualitative research. Newbury Park, CA: Sage. 1999; 163-177.

[8] Cakmak I, Kalayci M, Kaya Y, Torun AA, Aydin N, Wang Y (2010). Biofortification and localization of zinc in wheat grain. Journal of Agricultural and Food Chemistry..

[9] Lhussier M, Lowe N, Westaway E, Dykes F, McKeown M, Munir A (2006). Understanding communication pathways to foster community engagement for health improvement in North West Pakistan. BMC Public Health..

[10] Lowe NM, Westaway E, Munir A, Tahir S, Dykes F, Lhussier M (2015). Increasing awareness and use of iodised salt in a marginalised community setting in North-West Pakistan. Nutrients..

[11] Khan MU, Ahmad A, Aqeel T, Salman S, Ibrahim Q, Idrees J (2015). Knowledge, attitudes and perceptions towards polio immunization among residents of two highly affected regions of Pakistan. BMC Public Health..

[12] Khan A, Shaheen MA, Knight F, Baldi G (2017). Filling the nutrient gap in Pakistan: insights to address malnutrition. Nutrition Exchange..

[13] Birol E, Meenakshi JV, Oparinde A, Perez S, Tomlins K (2015). Developing country consumers’ acceptance of biofortified foods: a synthesis. Food Security..

[14] De Steur H, Wesana J, Blancquaert D, Van Der Straeten D, Gellynck X (2017). Methods matter: a meta-regression on the determinants of willingness-to-pay studies on biofortified foods. Annals of the New York Academy of Sciences..

[15] Olum S, Gellynck X, Okello C, Webale D, Odongo W, Ongeng D (2018). Stakeholders’ perceptions of agronomic iodine biofortification: a SWOT-AHP analysis in Northern Uganda. Nutrients..

[16] TRUST Consortium (2018) Global code of conduct for research in resource-poor settings, available at: http://www.globalcodeofconduct.org/. .

